# Aflatoxin Regulations in a Network of Global Maize Trade

**DOI:** 10.1371/journal.pone.0045151

**Published:** 2012-09-25

**Authors:** Felicia Wu, Hasan Guclu

**Affiliations:** 1 Department of Environmental and Occupational Health, University of Pittsburgh, Pittsburgh, Pennsylvania, United States of America; 2 Department of Biostatistics and Public Health Dynamics Laboratory, University of Pittsburgh, Pittsburgh, Pennsylvania, United States of America; Universidad Veracruzana, Mexico

## Abstract

Worldwide, food supplies often contain unavoidable contaminants, many of which adversely affect health and hence are subject to regulations of maximum tolerable levels in food. These regulations differ from nation to nation, and may affect patterns of food trade. We soughtto determine whether there is an association between nations' food safety regulations and global food trade patterns, with implications for public health and policymaking. We developed a network model of maize trade around the world. From maize import/export data for 217 nations from 2000–2009, we calculated basic statistics on volumes of trade; then examined how regulations of aflatoxin, a common contaminant of maize, are similar or different between pairs of nations engaging in significant amounts of maize trade. Globally, market segregation appears to occur among clusters of nations. The United States is at the center of one cluster; European countries make up another cluster with hardly any maize trade with the US; and Argentina, Brazil, and China export maize all over the world. Pairs of nations trading large amounts of maize have very similar aflatoxin regulations: nations with strict standards tend to trade maize with each other, while nations with more relaxed standards tend to trade maize with each other. Rarely among the top pairs of maize-trading nations do total aflatoxin standards (standards based on the sum of the levels of aflatoxins B_1_, B_2_, G_1_, and G_2_) differ by more than 5 µg/kg. These results suggest that, globally, separate maize trading communities emerge; and nations tend to trade with other nations that have very similar food safety standards.

## Introduction

Multiple nations worldwide have set food safety standards for maximum tolerable levels of certain contaminants in food, for the purpose of protecting public health. These standards, in turn, have important impacts on world food trade. Of interest is whether there is an association between nations' food safety regulations and global food trade patterns. For example, do nations with strict food safety standards tend to trade more with each other, while nations with more relaxed food safety standards also tend to trade with each other? Do the main food exporters tend to have stricter or more relaxed food safety standards themselves?

We are interested in these questions from the perspective of how food safety regulations that national governments impose may or may not have impacts on a global level, and what the implications might be for food safety and policy decision-making. Hence, we developed a network model to represent the global trade patterns of maize from nation to nation. We examined these maize trade patterns as function ofaflatoxin regulations in maize, with a focus on regulations for maize intended for human consumption, for each nation.

### Aflatoxin: Background

Aflatoxins are secondary metabolites of the common foodborne fungi *Aspergillusflavus* and *A. parasiticus*, which colonize crops in tropical and subtropical regions worldwide. These fungi can also produce aflatoxin in storage, transportation, and food processing. Aflatoxin contamination primarily occurs in maize, spices, peanuts, tree nuts (almonds, pistachios, hazelnuts, pecans, and Brazil nuts), and milk.

Aflatoxin B_1_, the most toxic aflatoxin, is the most potent naturally occurring chemical liver carcinogen known. The risk of liver cancer in individuals exposed to chronic hepatitis B virus (HBV) infection and aflatoxin may be up to 30 times greater than the risk in individuals exposed to HBV of aflatoxin alone [1]. Acute aflatoxicosis, causing severe gastrointestinal symptoms and often death, results from high aflatoxin doses. In recent years, hundreds of aflatoxicosis cases in Africa have resulted from consumption of contaminated maize [2]. Aflatoxin exposure may also be associated with stunting in children [3], [4] and immunosuppression [5]. Recently, aflatoxin exposure has been associated with liver cirrhosis; aflatoxin and HBV exposure may synergize to substantially increase cirrhosis risk [6].

Currently, over 5 billion people worldwide are at risk of chronic exposure to aflatoxin in food [2]. Maize is one of the main sources of human exposure to aflatoxin, because it is highly consumed worldwide and unfortunately is also one of the most susceptible crops to aflatoxin contamination [2], [7].

### Aflatoxin regulations worldwide and their potential impacts

Over 100 nations have set regulatory limits on allowable aflatoxin levels in human food or animal feed. Most of these nations regulate the sum of the levels of the four most prominent types of aflatoxins in food: B_1_, B_2_, G_1_, and G_2_. Hence, in our paper, “aflatoxin” is meant to refer to the sum of these aflatoxins unless otherwise specified.

In industrial nations, aflatoxin contamination in food primarily inflicts economic rather than health burdens. It reduces the price paid for crops, and can cause disposal of large amounts of food. Losses from aflatoxin in the US – in the hundreds of millions USD annually – are associated with market loss rather than health effects [9], as enforcement of aflatoxin standards and aflatoxincontrol methods have largely eliminated harmful exposures in food. In low-income nations, however, health impacts of aflatoxin are more severe. Many individuals are not only malnourished but also chronically exposed to high aflatoxin levels primarily through the staple foods of maize and peanuts, resulting in deaths from aflatoxicosis and liver cancer. Low-income nations often lack the resources, technology, and infrastructure necessary for routine food monitoring and aflatoxin control. Aflatoxin exposuresare typically highest in sub-Saharan African and Asian nations [7]. Further complicating the problem is that for a given level of aflatoxin exposure, cancer risk is more severe in low-income nations than in the industrial world because of higher HBV prevalence [1].

Globalization of food trade has exacerbated aflatoxin-related losses in three unfortunate ways:

1. Strict aflatoxin standards mean that many nations will export their best-quality foods and keep contaminated foods domestically, resulting in higher aflatoxin exposure in low- or middle-income nations where hepatitis prevalence is high.
2. Even the best-quality foods produced in some nations may be rejected for export because of aflatoxin levels exceeding the tolerable limit, resulting in millions of dollars in losses.
3. The cost of a rejected food shipment is substantial (about $10,000 per lot in demurrage fees, [8]), even if the lot can be returned to the country attempting to export.

These dilemmas led former United Nations(UN) Secretary-General Kofi Annan to recognize the magnitude of the problem of setting appropriate aflatoxin standards worldwide. He commented, “The EU [European Union] regulation on aflatoxins costs Africa $670 million each year in exports. And what does it achieve? It may possibly save the life of one citizen of the EU every two years. Surely a more reasonable balance can be found” [9]. Annan had based his statement upon the report of the Joint FAO (Food and Agriculture Organization)/World Health Organization (WHO) Expert Committee on Food Additives' 49^th^ meeting on aflatoxin, which assessed the effect of aflatoxin regulations on liver cancer depending on HBV prevalence [10], [11]. Joint FAO/WHO Expert Committee on Food Additives (JECFA) developed two scenarios, to determine the effect of moving from an enforced aflatoxin standard of 20 µg/kg(or 20 ng/g) to 10 µg/kg, in two hypothetical nations: one with HBV prevalence of only 1%, and another with HBV prevalence of 25%. In the first nation, tightening the aflatoxin standard yielded a drop in the estimated population risk of 2 additional cancers per year per billion people. In the second nation, tightening the aflatoxin standard for this population yielded a drop in the estimated population risk of 300 additional cancers per year per billion people.

Hence, in rich food-importing nations with low HBV prevalence, tightening the aflatoxin standard would reduce cancer risk by an amount so small as to be undetectable by epidemiological methods. But food-exporting regions with *high* HBV incidence – China, Southeast Asia, and Africa – could have greater health risk due to stringent aflatoxin standards. Until aflatoxin control methods become available and affordable, strict standards would encourage exportation of their best crops to preserve export markets. The poor-quality crops would be left for domestic consumption, inadvertently increasing liver cancer risk among HBV-infected populations [9].

The other aspect to former UN Secretary-General Annan's statementconcerns the purported adverse economic impacts to Africa of attempting to trade with the European Union. This statement about economic loss was based on estimates in Otsuki et al. [12], who developed an economic model of expected aflatoxin contamination in African crops and how much of their export market would thus be lost. But are African nations in fact trading much food with the EU at all? Or do these nations as well as other food-producing nations worldwide tend to export more of their food to nations that have more relaxed food safety standards? According to a 2005 World Bank report [13], the losses suffered by African nations attempting to export foods to the EU was not nearly as severe as had been predicted in [12], and the African shares for certain foods (dried fruits) actually increased. Wu [9] also estimated a much lower loss to African nations from the EU aflatoxin regulations, and hypothesized that this was because food trade patterns between Africa and the EU were not on such a large scale as estimated by [12].

These food trade patterns will have a very important impact on the sustainability of nations that rely upon food exports for their market economies, and may also have impacts for global public health. It is for the purpose of answering these questions, and exploring the nature of food safety regulations and potential impacts on food trade and global health, that we have developed a social network model of global maize trade to facilitate understanding of this association.

### Social networkmodels and their applications

Social network models have been used in public health research to explain and predict a variety of phenomena, includingthe spread of infectious disease and how to control that spread [14], how to control disease spread [15], and patterns of obesity and smoking prevalence in social circles of friends, family members, co-workers, and acquaintances ([16], [17], see also [18]). In the field of mycotoxins, a network analysis has been done on contaminants reported through the EU Rapid Alert System for Food and Feed (RASFF) by nation [19]. However, the history of social network modeling extends as far back as the early 1900s, when mathematical models described malaria transmission and a threshold level for the *Anopheles* mosquitoes that transmit the disease [20]. Is it pointed out in [21] that social network models enable us to understand behaviors at both individual and population/global levels in a way that simple random sampling cannot do, because random sampling removes individuals (in the case of this work, individual nations) from the social context that may influence their behavior.

## Methods

We developed a social network model of world food trade, focusing on maize because of its importance to populations' diets worldwide, large volume of trade worldwide, and propensity to be contaminated with aflatoxin. Each nation is represented as an individual node or “actor” in the model, connected to each other in pairs by flows of imported and exported maize. To quantify the relative importance of maize trade patterns between nations, we used the United Nations Commodity Trade Statistics Database (UN Comtrade, comtrade.un.org), gathering country codes and analyzing total maize exports and imports from and to each of these nations (to and from every other nation) for each year from 2000 to 2009. We summedmaize trade data (exports and imports) on a nation-by-nation basis for the years 2000 to 2009.

Then we converted these global maize trade data into a weighted and directed network model in the software program Pajek^TM^ [22], in which each nation is represented as a node, and the edges (or lines) are export/import connections between the countries with weights (represented by thickness of the arrows in the network diagram) equal to amount of maize traded. The size of each node is proportional to the square root of the total amount of maize exportedby that nation from 2000 to 2009, for ease of visualization. The direction of the edges, or arrows, denotes the direction of maize trade: each arrow between two nations emerges from the nation exporting the maize, and points to the nation importing the maize. The “distance” between all trading pairs of nations is then minimized in the program by using a force-based layout so that the network representation reveals *clusters*: groups of nations that tend to trade large amounts of maize amongst each other, which appear to form a sub-network within the larger network.

We also calculated the *degree* (of connectedness) of each node (e.g., of each nation). The degree is the number of edges connected to the node. In the network model of maize exports, the degree (rather out-degree) represents the number of other nations with which one nation has *exported* any maize from 2000–2009. Degrees and clustering patterns, taken together, are important in maize trade networks to understand how vulnerable (or resilient) nations would be in the event of food shortage elsewhere in the world. If a nation that exports maize has a high degree, then any issues that alter maize availability in that nation would potentially affect many other nations. These other nations, in turn, are even more vulnerable if they do not regularly import maize from many, if any, other nations worldwide. On the other hand, if an *importing* nation has a high degree (rather in-degree), this means that it is importing maize from many different nations. Thus, any failure to produce maize at expected levels in one of those nations would not necessarily jeopardize maize supplies in the importer.

In addition to analyzing which nations export and import maize, and with whom, we compiled information about aflatoxin regulations in nations worldwide. These regulations were taken from the Food and Agriculture Organization [23] report on mycotoxin regulations worldwide. Because the network model is specific to maize trade, we only includedaflatoxin standards relevant to maize in our database. For example, if a nation such as Kenya has an aflatoxin standard listed for peanuts but not for maize, then the nation is coded as not having set an aflatoxin standard for maize.

We examined whether nations trading maize had similar or dissimilar aflatoxin regulations, to understand the ease or difficulty of exporting nations to provide maize that had sufficiently low aflatoxin levels. The aflatoxin regulations are the basis for another network model representation, in which the maize trade patterns are recreated, but thenode sizes are made equal and are color-coded based on the relative strictness of aflatoxin regulations. Again, when distances between trading pairs of nations are minimized, the proximity of nodes reveals whether nations that have similar aflatoxin standards are clustered together in maize trading patterns.

## Results

Table 1 contains data on the top 20 maize-exporting nations and the top 20 maize-importing nations, as well as the total amount of maize traded in metric tons from 2000 to 2009. As Table 1 shows, the United States is by far the largest exporter of maize worldwide, with over half a billion metric tons of maize exported to other nations in the past decade. This amount exceeds the next largest exporter's total trade amount by over four-fold. Argentina and Brazil, two neighboring South American nations, are also large exporters. China is the largest Asian maize-exporting nation, while France and Hungary, despite their relatively smaller geographic size compared with other main exporters, also export large quantities of maize. South Africa is the largest African maize-exporting nation. Among countries that import maize, Japan is the largest maize-importing nation. The Republic of Korea, Mexico, Egypt, Taiwan, and Spain have also imported large quantities of maize over the last decade.

**Table 1:** Top maize exporting and maize importing nations worldwide, based on volume of trade from 2000–2009.

Rank	Top maize exporting nations and total amount exported 2000–2009, MTs		Top maize importers and total amount imported 2000–2009, MTs	
1	USA	526,670,541	Japan	170,279,244
2	Argentina	123,527,253	Republic of Korea	90,841,881
3	France	71,269,591	Mexico	69,857,045
4	China	65,558,093	Egypt	51,446,403
5	Brazil	54,473,911	Taiwan	47,282,122
6	Hungary	28,557,159	Spain	45,302,592
7	Canada	23,311,927	USA	33,978,967
8	Ukraine	19,568,172	Netherlands	28,629,716
9	South Africa	15,021,879	Malaysia	27,703,058
10	Paraguay	12,051,097	Iran	27,178,624
11	Mexico	11,923,079	Colombia	26,821,972
12	India	11,738,537	Canada	26,012,453
13	Germany	10,400,097	Algeria	20,230,143
14	Serbia	6,797,441	Italy	16,678,997
15	Thailand	5,366,268	Germany	16,548,899
16	Romania	4,859,320	Israel	15,658,213
17	Switzerland	3,620,319	Saudi Arabia	15,613,125
18	Netherlands	3,601,194	Portugal	14,245,589
19	Austria	3,394,665	Morocco	14,083,900
20	Bulgaria	2,962,606	United Kingdom	13,815,724

Source: United Nations Commodity Trade Statistics Database (UN Comtrade, comtrade.un.org). MTs  =  metric tonnes.

An interesting facet of these trade statistics is that several countries that are among the top 20 maize exporters are also among the top 20 maize importers. These include the United States, Canada, The Netherlands, Mexico, and Germany. This may be for a variety of reasons, including that imported and exported maize may serve different destinations (e.g., food or feed), may be needed for different purposes at different times of the year, and may reflect policies of individual agreements among grain companies in different nations. In fact, the United States and Canada have established a trade relationship in which they each have exported and imported large amounts of maize to each other over the last decade.

Table 2 lists the 20 exporting nations with the highest out-degree (i.e., the number of nations to which it has exported at least one consignment of maize from 2000–2009) and the 20 importing nations with the highest in-degree (i.e., the number of nations from which it has imported maize). Not surprisingly, as Tables 1 and 2 show, many of the top maize exporters worldwide are also the nations that export maize to the largest total number of nations. Again, the United States has the largest degree, exporting maize to 181 different nations worldwide from 2000 to 2009. Likewise, Argentina, South Africa, France, Canada, and Brazil also export maize to a large number of nations.

**Table 2:** Nations with highest degrees of maize exports and imports: number of other nations with which it trades.

Rank, by degree	Maize-exporting nations and total number of nations to which they export		Maize-importing nations and total number of nations from which they import	
1	USA	181	France	69
2	Argentina	150	Germany	66
3	South Africa	128	USA	66
4	France	122	Netherlands	62
5	Canada	108	Canada	58
6	Brazil	101	Italy	57
7	China	95	United Kingdom	56
8	Italy	94	Spain	53
9	Netherlands	86	Egypt	53
10	India	85	Switzerland	51
11	Australia	78	Turkey	47
12	Ukraine	72	Austria	46
13	Hungary	72	Saudi Arabia	46
14	Thailand	70	Russian Federation	44
15	Spain	70	South Africa	44
16	Germany	69	United Arab Emirates	44
17	Turkey	69	Bulgaria	43
18	United Arab Emirates	66	Israel	41
19	United Kingdom	63	Romania	40
20	Chile	60	Belgium	40

Source: UN Comtrade.

However, although Japan, Korea, and Mexico are the largest maize-importing nations, they are not included in the 20 nations that import maize from the largest number of nations as indicated by degree (Table 2, right columns). The implication is that they are importing large amounts of maize from a small number of countries. Also of interest is that many European nations are included in both columns, such as France, Germany, Italy, The Netherlands, Spain, and the United Kingdom. They conduct a substantial amount of maize trade amongst themselves within Europe.

**Figure 1:**
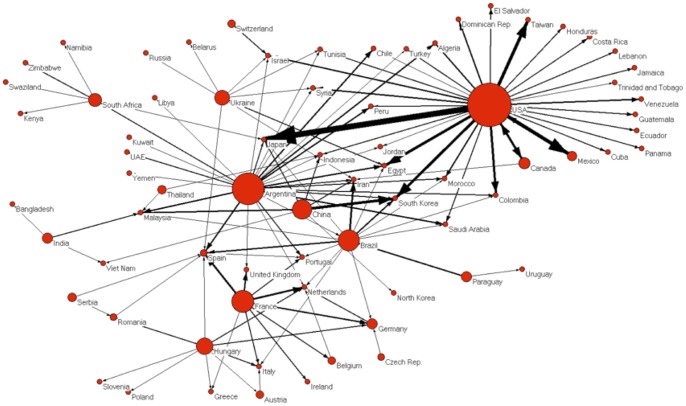
Global maize trade network emphasizing top exporters. The circle sizes are loosely proportional to the amount of maize exported. Each line represents export/import amount greater than 1 million metric tons from 2000–2009 [Wu and Guclu, unpublished data].

The social network model of maize trade between and amongst nations is shown in Figure 1 (export volume represented roughly by size of nodesfor each nation [Wu and Guclu, unpublished data]). An edge is drawn between two nations if they had traded more than one million MTs total in the years 2000 to 2009. The direction of the arrows indicates the direction of the maize trade: from an exporting nation to an importing nation.

Figure 1 shows that at least two distinct clusters emerge when distances are minimized among all trade partnerships: European nations in one cluster of inter-trade (lower left portion of Figure 1), and the United States and other American nations in another such cluster (upper right portion of Figure 1). Meanwhile, Argentina, China, and Brazil are at the center of the network: China exporting maize to Asian nations, and Argentina and Brazil trading with multiple different nations across the world.

In the maize trading network, the United States is at the center of a star-shaped topology. It exports large quantities of maize to a large number of nations, many of which do not import significant amounts of maize from any other nations. Hence, if any circumstance jeopardized the amount of maize that the US could afford to export in any given season, some countries could experience a substantial loss in maize supply – particularly nations in Latin America. This was particularly relevant in early years of large amounts of US maize being directed to ethanol production, and impacts on other nations [24]. However, other countries that import large amounts from the US (in the Middle East and Asia) are importing maize from other parts of the world as well.

European nations trade much of their maize amongst each other; from the outside, several of these nations also import maize from Brazil and Argentina. However, none of these European nations imported more than one million MTs of maize over ten years from the United States, despite the extremely large volume of maize exports from the US. On the whole, this portion of the maize trading network appears more stable; as a relatively smaller number of nations are receiving most of their maize imports from just one nation.

Many of the largest maize importers source their maize from multiple different parts of the world, such as Japan and Korea (importing from the United States, Brazil, Argentina, China, Israel, and South Africa) and several European nations. However, Mexico, though a large maize importer, purchases almost all of its maize from just one nation: the United States. This is also the case with many other nations in the Americas.

**Figure 2:**
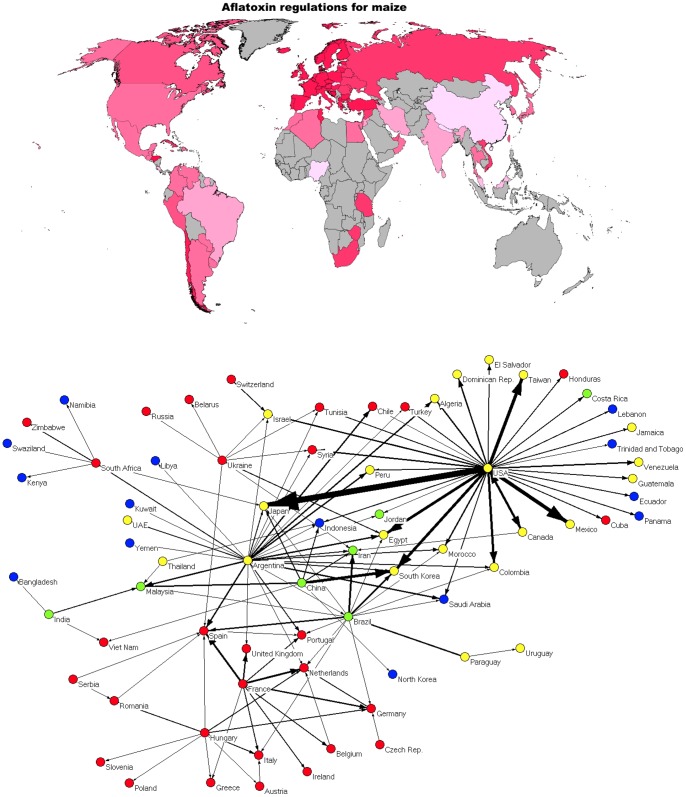
Color-coded maximum aflatoxin levels in maize by country: a) On the world map, and b) on the trade network. Each edge in 2b represents an export/import amount greater than 1 million metric tons from 2000–2009.

Figures 2a and 2b depict the aflatoxin standards for maize set by each individual nation around the world. In Figure 2a, darker hues represent nations with stricter aflatoxin standards, while nations in gray have not yet set maximum allowable standards for aflatoxin in maize. Figure 2b is the maize network representation with colors indicative of the relative stringency of the aflatoxin standard for maize.

Figure 2b shows that among the maize-trading clusters identified previously, aflatoxin regulations look very similar. It is not surprising that the European maize trading community in the lower left is homogenously colored, as the EU has set aflatoxin standards that apply across all member states [25]. This EU standard is relatively strict compared with other parts of the world. However, it is interesting that the United States, which has a relatively relaxed total aflatoxin standard of 20 µg/kg (“total aflatoxin” refers to the sum of the levels of aflatoxins B_1_, B_2_, G_1_, and G_2_: the four major types of aflatoxins), primarily exports maize to other nations that also allow relatively larger amounts of aflatoxin in maize. There are, however, several exceptions: several Latin American and Middle Eastern nations that have strict aflatoxin standards (Honduras, Cuba, Chile, Turkey, Tunisia, and Syria) import large amounts of maize from the US. Notably, African nations do not export substantial amounts of maize to the EU. Several sub-Saharan African nations are in a maize trading cluster in the upper left of Figure 2b, while northern African nations such as Egypt, Morocco, and Tunisia are more in the center of the global trading pattern.

These trends are highlighted as well in Table 3, which lists the 20 pairs of nations that have engaged in the greatest volume of maize trade in the last ten years, the aflatoxin standards in these nations (compiled from [23]), and the total amount of maize traded from 2000–2009. Nations are not included in this table if they have set aflatoxin standards for other foodstuffs such as peanuts, but not for maize. In some nations, the aflatoxin standard is set specifically for aflatoxin B_1_, rather than for total aflatoxins. To extrapolate to an estimate of total allowable aflatoxins, we multiplied the maximum allowable concentration of aflatoxin B_1_ by two.

**Table 3:** Top volumes of maize trade worldwide from 2000–2009.

Rank	Top exporter-importer pairs and their total aflatoxin (AF) standards in µg/kg maize				Total amount (MT)
	Exporter	AF standard	Importer	AF standard	
1	USA	20	Japan	20	159,377,000
2	USA	20	Mexico	20	69,764,700
3	USA	20	Taiwan	15	44,212,000
4	USA	20	Korea	20	41,657,300
5	China	40	Korea	20	36,446,400
6	USA	20	Egypt	20	35,540,100
7	USA	20	Canada	15	25,933,000
8	USA	20	Colombia	20	21,726,900
9	Canada	15	USA	20	21,161,900
10	France	4	Spain	4	18,682,400
11	France	4	Netherlands	4	14,901,600
12	Brazil	30	Iran	30	12,588,000
13	Mexico	20	USA	20	10,947,000
14	Argentina	20	Chile	5	10,625,700
15	USA	20	Algeria	20	10,457,700
16	USA	20	Dominican Rep.	20	10,325,300
17	Argentina	20	Spain	4	10,311,600
18	China	40	Malaysia	35	10,119,800
19	France	4	UK	4	9,899,890
20	Argentina	20	Egypt	20	9,734,360

Source: UN Comtrade and FAO [22]. MT  =  metric tonnes.

Among these major maize trade relationships, there are but a few instances in which an exporting nation trades maize to an importing nation with a significantly different aflatoxin standard. In general, aflatoxin regulations in two nations that trade maize with each other do not differ by more than 5 µg/kg. In fact, in the majority of these top 20 trading relationships, the importing and exporting nations have the same aflatoxin standard for maize.

## Discussion

Earlier social network analyses in public health have demonstrated that “like attracts like.” Controlling for other socioeconomic and demographic factors, smokers tend to be in closer social bonds with other smokers [17], and obese individuals form closer bonds with other obese individuals [16]. The same appears to be true of nations that trade maize with each other and the relative strictness of food policies that pertain to maize in individual nations. Namely, nations that share strong food trade ties tend to have similar regulations on allowable levels of aflatoxin in maize.

Certain trade clusters for maize clearly emerge. The United States is the largest exporter of maize in the world, and occupies a corner of the maize trade network that places it in the center of a cluster of nations that import maize almost solely from it. Most US maize trade is done in with Canada, Latin American nations, and Middle Eastern nations; it does not export much maize at all to Europe. Notably, the aflatoxin regulations in the nations to which the US exports maize are roughly the same as the US Food and Drug Administration aflatoxinregulations.

European nations form another cluster of maize trade, completely separated from the US maize cluster (i.e., no direct links between the US and any European nation at the level of 1 million MTs maize traded from 2000 to 2009). In this European cluster, it is not surprising that most nations have the same aflatoxin regulation, as many of these nations are EU member states that share mycotoxin regulations [23]. Within this cluster, France and Hungary are the main maize exporters, while Spain and the Netherlands are main importers. In contrast to former UN Secretary General Annan's statement, because EU member states do not import much maize at all from Africa, it is unlikely that African maize exports would be adversely affected by the strict EU aflatoxin standard. Our findings here are in agreement with those of [9] and [13]. Other crop exports may be, however; which was not analyzed in this study.

In between these two distinct clusters are nations that export maize to multiple different parts of the world: Argentina, Brazil, and China. These three nations supply maize to Africa, the Americas, Asia, Europe, and the Middle East. Though they themselves have relatively relaxed aflatoxin standards, Argentina and Brazil export maize to multiple European nations with much stricter aflatoxin standards. However, all three nations trade more with other nations that have relaxed standards or no standards at all for aflatoxin in maize.

The largest importer in the world of maize isJapan; which, in an average year, imports nearly twice as much as the next largest maize-importing nation, Korea. Mexico, Egypt, and Taiwan are also large maize importers. Japan, Korea, and Egypt all import maize from at least three different continents; which makes them less vulnerable to maize scarcity if maize supplies for export become limited in one part of the world. Taiwan and Mexico, on the other hand, import almost all of their maize from the United States. Hence, their maize supply is heavily dependent upon continued availability of maize for export from the US. Possibly because they rely heavily on maize imports, all five of these nations have relatively relaxed standards for aflatoxin in maize.

These clustering patterns and directionality of maize trade have important implications for food security. If the members in a cluster are not well-connected, then the cluster may be more vulnerable to any adverse consequences that may affect the central nation in the cluster. Because the United States is at the center of a large cluster of North and Latin American nations, which themselves are hardly connected with each other or with other nations in the network, then this portion of the network of maize trade would become extremely vulnerable to reduced food supply and increased prices if anything should affect the quality or quantity of US maize available for export. This was relevant in the case of US maize ethanol production and its attendant effects on maize-importing nations in recent years. Because European nations are more densely clustered in maize trade and also source maize from Brazil and Argentina, they may be more stable to fluctuations in maize supply in any one part of the world.

One limitation of static network models is the inability to prove causality. We have shown that nations tend to cluster into maize trading communities that share similar aflatoxin regulations. However, do the regulations cause the trade patterns to emerge as they are, or do the trading patterns influence the regulations that nations set? Or might both be possible? Although causality for one of these cannot be proven, one future research direction involves temporal modeling of food trade and regulation enactment at different points in history, to examine whether the trade communities preceded the enactment of regulations, or *vice versa*.

If the specific trade community determines food safety regulations in multiple nations, then the implication is that certain central nations in the network have a large amount of power in determining regulations elsewhere worldwide. However, if regulations determine trade patterns, then nations should be aware of the implications their standard setting will have on who their future food trading partners worldwide will be. Because the implications are important for food security in ways that extend beyond aflatoxin exposure, this is an area of research that deserves further attention for the purposes of policy decision-making.
